# Stress Coping Strategies in Parents of Newborns and Infants with Congenital Cyanotic Heart Disease with Regard to Stress Levels and Negative Emotions

**DOI:** 10.3390/children11050508

**Published:** 2024-04-24

**Authors:** Agnieszka Kruszecka-Krówka, Grażyna Cepuch, Agnieszka Micek

**Affiliations:** 1 Nursing and Midwifery Institute, Faculty of Health Sciences, Jagiellonian University Medical College, 25 Kopernik Street, 31-501 Krakow, Poland; grazyna.cepuch@uj.edu.pl; 2 Statistical Laboratory, Faculty of Health Sciences, Jagiellonian University Medical College, 25 Kopernik Street, 31-501 Krakow, Poland; agnieszka.micek@uj.edu.pl

**Keywords:** infants, cyanotic heart defect, parents, stress, coping with stress

## Abstract

Background: Parents of children suffering from congenital heart disease experience high levels of stress and negative emotions. Therefore, recognition of parents’ emotional states and their ways of coping with it is becoming more and more important. Methods: The study group consisted of 154 parents of newborns and infants with cyanotic congenital heart disease, before and after cardiac surgery (partial or full). To assess parental negative emotions, the level of stress, and strategies of coping with it, standardized questionnaires, such as HADS-M, PSS-10, and COPE, were used. Results: Stress levels in parents were high and associated with negative emotions (anxiety, depression, irritability), as well as the choice of non-constructive coping strategies, which was observed especially in younger parents. Conclusions: Assessing parents’ stress levels and ways of coping with stress can improve family functioning and provide better development conditions for the child.

## 1. Introduction

Approximately 400,000 children are born in Poland every year [1]. Similarly to other countries, in Poland, heart defects are the leading cause of death in children born with congenital defects [1,2]. The problem persists despite an increase in the efficiency of diagnosing congenital defects in newborns and infants, an increase in the number of cardiac surgeries [1], improved treatment standards, and a general increase in survival rates in this group [3,4,5,6].

Encouragingly optimistic scientific reports in the field of pediatric cardiac surgery do not reduce parents’ fear, anxiety, and crisis situations related to the diagnosis of a heart defect in their child [7,8,9,10]. Parents may experience anxiety, depression, and toxic stress as early as the diagnosis of a defect in the prenatal period [11]. Negative emotional states can also determine parents’ psychological functioning and have an impact on their relationship with their child [12,13,14] and the child’s further development [14]. The role of stress, anxiety, and depression in parents cannot be ruled out as factors responsible for reducing therapeutic success and the dynamics of the child’s recovery. Considering the aforementioned factors, it becomes important to recognize parents’ emotional states and stress levels [15] and the ways in which they cope with stress in a difficult and unpredictable situation, such as when face with their child’s illness. It is important to bear in mind that the way of coping with stress depends mainly on individual predispositions, and the choice of desirable strategies depends on the assessment of a stressful situation [16,17]. The way parents cope with a critical situation is also determined by their adaptive capabilities in dealing with such circumstances [17,18,19]. It is important that their coping strategies of dealing with stress are flexible enough to enable the realization of both the goals related to the child’s illness and the general goals of each parent [20]. When faced with having a child with a severe congenital heart defect, the ability to apply a task-based approach to the disease may not only improve parents’ emotional functioning but also lead to beneficial changes in the entire family system [11].

Taking into account the emotional state of parents of children with congenital heart disease and their ability to cope with stress in the process of multidisciplinary treatment is an investment related not only to the better final therapeutic outcome for, functioning of, and future of the family, but also to the reduction of the costs associated with the therapeutic process of the child and social costs. Implementing measures that also focus on supporting the parents of the treated child will optimize and individualize the holistic efforts of the medical team.

In view of the above, an attempt was made to assess the stress coping strategies of parents of children with congenital cyanotic heart disease using screening tools that are easily accessible, inexpensive, and usable by members of medical teams who are not psychologists. The application of such tools can help to identify parents with problems without generating additional financial expenditures in the child’s treatment process.

The aim of this study was to evaluate selected coping strategies, taking into account the level of stress, anxiety, depression, and irritability/aggression in mothers and fathers of newborns and infants with congenital cyanotic heart disease at different stages of cardiac treatment. In the other scientific reports reviewed, the authors focused on parents, taking into account various variables, but did not include parents of children with severe heart defects, including defects without a chance of full correction. We focused exclusively on a narrow group in which a risk to the child’s life could be observed. We aimed to use tools that were simple and easy to use by parents and interpretable by any member of the treatment team. The research toolkit applied in our study was not used for such a group of parents in the reports published by other authors.

## 2. Materials and Methods

### 2.1. Study Design

This study conducted was a cross-sectional one. Its purpose was to diagnose the level of stress and the ways in which mothers and fathers of newborns and infants with congenital cyanotic heart disease cope with this situation. This study also included an assessment of the levels of anxiety, depression, and irritability/aggression in the study group of parents who accompanied their children during their stay in hospital. The STROBE checklist [21] was used in editing the report.

In order to achieve the assumed goal of the study, the following research questions were formulated:

1. What is the relationship between parents’ stress levels and anxiety, depression, irritability, and selected sociodemographic and clinical variables (time of defect diagnosis and stage of cardiac surgery treatment)?
2. What stress coping strategies do parents choose depending on their gender and age?
3. What is the relationship between parents’ choice of coping strategies and the level of stress and other variables studied?

### 2.2. Sample, Setting, and Data Collection

The survey was conducted among mothers and fathers of newborns and infants with cyanotic congenital heart disease at the Center for Cardiology and Pediatric Cardiac Surgery in southern Poland. The results in the current report are a continuation of an analysis from a study partially published in *Children* [11] that aimed to assess the prevalence of anxiety, depression, aggression, and levels of perceived stress in a group of parents of newborns and infants with cyanotic heart defects, both before cardiac surgery and after the procedure. The current report does not replicate the results of the previous study but focuses mainly on parents’ strategies of coping with stress, taking into account selected predictors.

The recruitment process selecting parents for the study took place in two stages. The first stage included a detailed analysis of the medical records of patients, based on which a group of newborns and infants with a cyanotic heart defect diagnosed in fetal life or immediately after birth was selected. From this group, parents were selected whose children were in stable health, without other concomitant defects or conditions, and who were at different stages of cardiac treatment (1. full correction of the heart defect; 2. partial correction of the heart defect, which meant one of the stages of cardiac treatment; 3. Waiting for correction of the heart defect).

The second stage of recruitment consisted of selecting from the shortlisted parents only those who met further inclusion criteria. The detailed inclusion criteria for the parents and the group of newborns and infants was presented in a scientific report published in *Children* [10]. After the previously conducted two-stage recruitment, the proper study was launched, which was conducted from January to May 2023.

Each parent qualified for the study, before deciding to participate, was given information about the purpose of the study, its course, and the possibility of withdrawing from the study at any stage without giving a reason. Parents gave their voluntary consent to participate in the study and to use the collected material for publication.

This study’s authors chose only fully completed questionnaires for further examination. Finally, questionnaires from 154 parents were statistically analyzed.

Every effort was made to preserve the privacy and confidentiality of the survey participants. In order to prevent the identification of children as well as their parents by anyone other than the authors of the survey, all information provided by respondents was coded and all non-numerical information was removed. The survey was conducted in accordance with the ethical principles of the Declaration of Helsinki. The course of the study was approved by the Bioethics Committee of the Jagiellonian University (No. 118.6120.86.2023).

### 2.3. Participants and Involvement

Newborns and infants were not directly involved in the study in any way, and the leading source of information was the parents of children who met the selection criteria.

### 2.4. Description of Research Tools

The research tools consisted of the following:

- Self-designed questionnaire, including sociodemographic data of parents and selected clinical variables relating to children [10].
- The Hospital Anxiety and Depression Scale (HADS) [22,23]. For the purpose of the current study, three subscales (anxiety, depression, and irritability/aggression) were analyzed separately as well as in total.
- The Perceived Stress Scale—10 (PSS-10) [24,25], which is used as a screening tool to assess the stress level and identify those in need of support. A high score on the PSS scale indicates that the assessed life situation is highly stressful, overburdening, and unpredictable.
- Coping Orientation to Problems Experienced (COPE) [25,26]. The COPE questionnaire includes 60 statements related to 15 strategies for responding to/coping with stressful situations and is based on Lazarus and Folkmans’ transactional model of stress Carver et al. [26]. The scale can determine someone’s primary coping styles with scores on the following three subscales: I. Problem-Focused Coping; II. Emotion-Focused Coping; III. Avoidant Coping [27]. The tool makes it possible to assess, based on dispositional coping, which of the given strategies are most often used in a stressful situation. The analysis of the responses given by the participants of the survey followed the key developed by the authors of the scale [25,26] and the study by Dias et al. [27]. In summary, a score (ranging from 4 to 16) was calculated for each of the 15 strategies as the sum of the 4 component statements. Then, the coping strategies thus obtained were grouped into 3 main subscales consisting of 5, 6, and 4 of the 15 strategies, respectively, and score values were summed within these groups. In addition, for the purpose of data analysis, each of the 15 strategies and the 3 main subscales were divided into high-level and low-level categories, with a cutoff point in the second tertile of the distribution of each variable.

### 2.5. Statistical Analysis

The characteristics of the study group were presented separately for parents with low or average levels of stress and those with high levels. Absolute and relative frequencies were used to describe individual qualitative characteristics, while distributions of variables of the quantitative type were presented as means and standard deviations (SD).

The significance of differences in demographic and clinical characteristics between groups with different levels of stress, as well as differences in stress coping strategies between parents of different sexes and ages, were compared using the chi-square test of independence and Student’s *t* test, after verifying the assumptions necessary for their application. In addition, Spearman rank correlations were calculated, which examined the association between the choice of stress coping strategies (score) and the level of stress (score) and the parents’ age. Finally, the stability of the preliminary results verifying the relationship between PSS and individual statements from the COPE questionnaire was verified in analyses including other variables characterizing the child and parents. Logistic regression analyses were conducted separately for each of the stress coping strategies and main subscales, as with dichotomous dependent variables (1—high level of a given COPE strategy, i.e., above the second tercile of the distribution; 1—low level of a given COPE strategy, i.e., below the second tercile of the distribution) and with the dichotomous variable PSS (1—high; 0—low or medium) as the main explanatory variable in the constructed models. Results were presented with odds ratios (ORs) and 95% confidence intervals (CIs). Analyses were performed in R, version 4.0.4 (R Foundation for Statistical Computing, Vienna, Austria). Standard two-tailed tests were used to determine significance at the level of 0.05.

## 3. Results

### 3.1. Characteristics of the Study Group

High levels of stress were observed in 117 parents (75.97%) (Table 1). Age, gender, place of residence, parents’ education, time of diagnosis of the child’s heart defect, and stage of cardiac surgery did not differentiate the subjects’ stress levels. However, parents with higher levels of stress scored statistically significantly higher on depression, anxiety, and irritability scales, as assessed by the HADS scale, and obtained a higher score for certain stress coping strategies, namely, 10. Focus on and venting of emotions, 11. Denial; 13. Behavioral disengagement, and 14. Substance use, and lower for 7. Positive reinterpretation and development.

### 3.2. Parents’ Coping with Stress by Gender, Age and Stress Level

Higher scores in 3. Seeking Instrumental Support and 15. Sense of Humor and subscale III. Avoidant Coping were obtained for mothers. The younger parents’ age categories had statistically significantly lower scores for 2. Planning, 7. Positive reinterpretation, and development, as well as subscale I. Problem-Focused Coping, while the oldest age category had a statistically significantly higher score for 10. Focus on and venting of emotions (Table 2).

In contrast, the PSS score was positively correlated with the choice of strategies 10. Focus on and venting of emotions, 12. Mental disengagement, and 14. Substance use and negatively correlated with the choice of strategies 7. Positive reinterpretation and development and 9. Acceptance. The correlation coefficients between the choice of stress coping strategies and the level of stress and parents’ age are shown in Table 3.

### 3.3. Coping with Stress and Selected Sociodemographic and Clinical Variables

**Figure 1:**
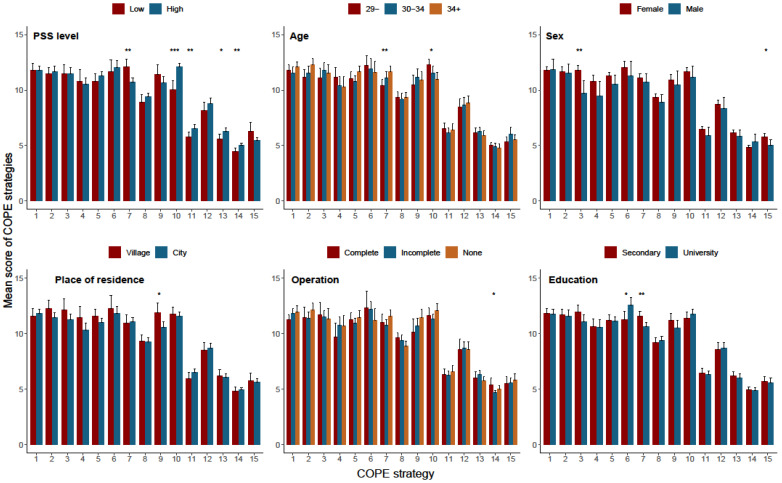
Association between high level of PSS and the choice of COPE strategies. Note: *** *p* < 0.001, ** *p* < 0.01, * *p* < 0.05. 1. Active coping; 2. Planning; 3. Seeking instrumental support; 4. Seeking emotional support; 5. Suppression of competing activities; 6. Turning to religion; 7. Positive reinterpretation and development; 8. Restraint coping; 9. Acceptance; 10. Focus on and venting of emotions; 11. Denial; 12. Mental disengagement; 13. Behavioral disengagement; 14. Substance use; 15. Sense of humor.

Logistic regression analysis showed that regardless of parents’ gender, age, education level, and place of residence, as well as the period of diagnosis of the child’s heart defect and the stage of cardiac surgery treatment, higher levels of stress were associated with increased odds of choosing certain COPE strategies, with odds ratios of 3.45 for 2. Planning; 4.17 for 10. Focus on and venting of emotions; 2.53 for 11. Denial; 2.72 for 13. Behavioral disengagement and 3.36 for 14. Substance use. At the same time, this group of parents showed an 81% reduction in the chance of choosing strategy 7. Positive reinterpretation and development (Table 4, Figure 1).

## 4. Discussion

The diagnosis of a severe congenital heart defect in a child is a factor that affects parents’ well-being, both physically and emotionally, regardless of the time of the diagnosis [8,10,28]. A wide range of stressors are associated not only directly with the child’s illness but also with financial problems (e.g., the burden of medical expenses) and occupational and/or relationship problems (feelings of loneliness, losing social contacts), which particularly affect mothers [29,30]. In response to persistent environmental stressors, parents can develop toxic stress syndrome [31,32,33], with adverse consequences for both themselves [34] and their children [35], which was confirmed by the survey results obtained.

As it was shown by the results of the current study, nearly 76% of parents reported high levels of stress, regardless of the dependent variables studied. An analysis of other reports in this area indicates similar results [36,37,38]. In view of the results of the current study and the cited reports [39,40], a careful assessment of stress in parents can be recommended, taking into account not only its level, but also its leading sources. In our study, the association of high levels of stress with depression, anxiety, and irritability is clearly indicated. It seems clear that special attention should be paid to pregnant women with a diagnosed fetal heart defect [10,38] because high levels of stress can affect not only the course of the pregnancy itself but also the course of the postpartum period. Biber et al. [41], in their review of studies on the psychological situation of parents of children with congenital heart defects, presented the extent of problems experienced primarily by mothers.

The effects caused by stress and negative emotions among parents of children with cyanotic heart defects negatively affect the functioning not only of the person who experiences them, but also of the entire family [11,12]. To reduce stress and cope with emotions, parents undertake various coping strategies, depending on individual predispositions and personal resources. Parents participating in the study who showed high levels of stress correlated with negative emotion focused on their emotions, denied the situation, gave up making efforts to achieve a goal, and were more likely to use stimulants to temporarily alleviate unpleasant emotions. If such a group of parents is detected by a medical team who are not psychologists, especially while diagnosing a defect in a child in the prenatal period, [42], this will allow for early support measures to be taken [14], and in the case of parents in a particularly difficult emotional situation, this will allow them to be referred for professional psychological support. This is important because reports indicate that stress persists for a long time with an upward tendency, even until the child reaches preschool age [43,44].

Roberts et al. [9] in their report emphasize the importance of parents’ emotional state for the long-term neurodevelopmental outcomes achieved by a child with congenital heart disease. Despite other tools used by the aforementioned authors, there is a clear relationship between low parental stress and good stress coping and a constructive approach to the task assessed by the authors (the secondary control engagement coping was associated with lower parent total stress).

An attempt undertaken in the current study to assess parents’ stress coping strategies confirmed that mothers were more likely to seek instrumental support and to neutralize stress through humor. At the same time, mothers were characterized by choosing avoidant strategies. Similar results were obtained by Choi and Lee [45], showing, among other things, a relationship between high levels of stress and self-efficacy in coping with stress and opportunities for support. At the same time, Choi and Lee [45] put forward a thesis that perhaps for mothers, social and not necessarily instrumental support is one of the most important elements in coping with stress (mothers’ tendency to rely on coping strategies using social support). Also, a report by Jackson et al. [46] indicates a relationship between coping skills and a reduction in perceived stress. At the same time, support, especially based on the individualized needs of the recipients, is extremely important. Similarly, Miller et al. [47] showed that improving social interactions (e.g., using mobile apps) promotes better coping with stress among parents. Dardas and Ahmad [48] demonstrated that the coping strategies of seeking social support and avoiding escape acted as a buffer correlated with lower stress levels. As reported by Poh et al. [49], good communication and the integration of the family become crucial. Admittedly, the cited study focused only on mothers and their readiness for their child being discharged from hospital, and the researchers used different tools compared to those used in the current study, but based on the results presented, it can be concluded that the level of stress is a significant predictor of the choice of coping strategies, determining parents’ behavior.

In turn, Fairfax et al. [50] pointed out the problem of coping with difficult situations in relation to the quality of life of seriously ill children’s parents. By analyzing scientific reports in this area, they hypothesized that positive or adaptive coping strategies may be positively related to parents’ psychological quality of life.

It is difficult to compare the results obtained in the current study with those of other authors and reach explicit conclusions because the scientific reports analyzed used questionnaires for assessing coping with stress that were different from those used in our own study. The possible discrepancy in the obtained results and the need to interpret them cautiously in the explored subject area were also pointed out by Egleson et al. [51], emphasizing the need to standardize research tools.

Interesting results were also obtained by Mussatto et al. [30], proving that stress, its level, and coping factors were more significant predictors of well-being than demographic variables. In contrast, the results of the current study indicate that a demographic variable such as parental age is a significant variable in how parents cope with stress. Younger parents were less likely to focus on planning, positive reevaluation of situations, and their own development. In general, they were less likely to choose solution-focused strategies. At the same time, it should be noted that regardless of gender, age, level of education, and parents’ place of residence, as well as the period of diagnosis of the child’s heart defect and stage of cardiac surgery treatment, higher levels of stress were associated with a greater chance of choosing coping strategies such as planning, focusing on emotions, denial, suppression of competing activities, and use of stimulants. While planning can be considered a positive strategy, the others are undesirable ones and can destabilize the functioning of the individual and their family. According to the interpretation of the COPE questionnaire, planning is a consideration on coping with a stressor, which, in the context of the current study, is the child’s illness. It is difficult to explain such choices, given the inability to compare them with other reports. Perhaps the choice of planning strategy is a result of the need for parents to make a number of key decisions related to their child’s illness and treatment, such as agreeing to diagnostic tests, cardiac surgery, invasive and non-invasive procedures, or participation in nursing.

Unfortunately, due to a wide variation in the research tools used, comparing results can contribute to discrepancies in the interpretation of the reports [52]. In interpreting the results of different authors, as well as in developing support models for parents, the demographic and cultural characteristics of the groups studied should also be taken into account.

### 4.1. Implications

In view of a wide spectrum of emotional dysfunctions and problems faced by parents of children with congenital heart disease, it becomes important to identify priority psycho-emotional problems by implementing routine screening [53,54]. The Stress (PSS-10) and Anxiety, Depression, and Distress (HADS-M) assessment tools used in the current study are widely available and easy for professionals who are not psychologists to use and interpret. In addition, these tools are widely used in research, making it possible to compare and interpret the results obtained. On the other hand, The Coping Orientation to Problems Experienced (COPE) questionnaire has similar qualities to the PSS-10 and HADS-M, but an analysis of scientific reports did not provide comparative material from the area we studied. In the opinion of the authors, the COPE questionnaire was well received by the surveyed parents, easy to use, and not controversial, providing an opportunity to reliably assess the stress coping strategies used. The results obtained with this tool enable medical teams to develop optimal and individualized support models for parents.

In view of the results obtained from the current survey, the group that requires special attention is young parents. Although the survey was conducted in a single center, it can be considered a study that gives an overview of the situation of parents throughout the country. Poland is a homogeneous and consistent country in many respects (e.g., language, accessibility to medical treatment, methods of treatment, and education). In Poland, there are no privatized pediatric cardiac surgery centers or centers providing treatment to selected groups of the population, and the level of treatment is homogenous, so these variables do not affect the results obtained. Each of only a few paediatric cardiac surgery centers in Poland accepts patients from the whole country (there is no regionalization). Given this information, a single-center survey of such a number of respondents can be considered representative of the entire group of parents of children with such heart defects, and similar education and support programs can be applied for all of them.

It should be noted, however, that due to increased social migration, including an influx of people from other countries, the cultural factor would seem to be important in the next planned scientific studies. Due to the above limitations of this study, the obtained results should be interpreted carefully.

### 4.2. Strengths and Limitations

The presented study has some limitations that should be taken into account when interpreting the results and planning future research in this area. First of all, the study group was dominated by women, with a significant predominance of mothers of newborns diagnosed with prenatal heart defects. Therefore, it would be beneficial to expand the study to include other centers, including those outside southern Poland, with a greater focus on the fathers and parents of newborns with a heart defect diagnosed postnatally. In addition, the study did not take into account economic variables and whether parents had children other than the patient, which may have determined the choice of stress coping strategies in the first place. Moreover, the assessment of emotional state, stress, and coping strategies was carried out at only one time point. Arguably, it would make sense to examine the trajectory of stress and negative emotions, as well as the choice of coping strategies at different stages of treatment and the child’s development. It would also be valuable to consider available sources and types of support, which may be potential predictors of anxiety, depression, irritability, and stress levels, as well as coping styles and strategies presented. In the context of planning and conducting further research in this area, it is important to take into account cultural factors associated with increasing social migration.

## 5. Conclusions

The levels of stress in the group of parents of children with congenital heart disease were high. Higher levels of stress were associated with higher levels of anxiety, depression, and irritability, as well as the selection of non-constructive coping strategies, such as mental disengagement, substance use, and focus on and venting of emotions, especially in the case of younger parents.

Mothers were more likely than fathers to seek instrumental support and resort to a sense of humor. The time of diagnosis of the heart defect and the stage of cardiac surgery did not differentiate the subjects’ stress levels. Higher levels of stress were associated with an increased chance of choosing certain coping strategies, regardless of sociodemographic and clinical variables.

Assessing the level of stress and ways to cope with it allows for the construction of optimal models of medical and psychological care to minimize family burdens and improve parental functioning, thus achieving better developmental outcomes in children.

## Figures and Tables

**Table 1:** Characteristics of the study group across categories of stress level.

Variable	PSS		*p*
	Low/Average (*N* = 37)	High (*N* = 117)	
Child’s (clinical) characteristics			
Correction, *n* (%)			
Full	5 (13.51)	19 (16.24)	
Partial	19 (51.35)	63 (53.85)	0.814
No correction	13 (35.14)	35 (29.91)	
Time of defect diagnosis, *n* (%)			
Prenatal	27 (72.97)	84 (71.79)	1.000
Postnatal	10 (27.03)	33 (28.21)	
Respondent’s (parent’s) characteristics			
Sex, *n* (%)			
Female	30 (81.08)	102 (87.18)	0.513
Male	7 (18.92)	15 (12.82)	
Age, mean (SD)	32.84 (5.03)	31.74 (4.87)	0.246
Age, *n* (%)			
≤29	8 (21.62)	43 (36.75)	
30–34	15 (40.54)	38 (32.48)	0.234
≥35	14 (37.84)	36 (30.77)	
Residence, *n* (%)			
Village	9 (24.32)	26 (22.22)	0.967
City/town	28 (75.68)	91 (77.78)	
Education, *n* (%)			
Elementary/vocational/secondary	23 (62.16)	51 (43.59)	0.075
High	14 (37.84)	66 (56.41)	
HADS D score, mean (SD)	6.32 (3.59)	10.6 (2.92)	<0.001
HADS A score, mean (SD)	10.14 (4.14)	12.69 (2.93)	0.001
HADS I score, mean (SD)	2.7 (1.18)	3.67 (1.33)	<0.001
Strategies (COPE)			
1	11.81 (1.81)	11.78 (1.97)	0.925
2	11.49 (1.66)	11.68 (2.6)	0.589
3	11.49 (2.55)	11.5 (2.88)	0.985
4	10.76 (3.39)	10.54 (3.23)	0.731
5	10.76 (2.22)	11.28 (1.94)	0.201
6	11.65 (3.26)	12.01 (3.46)	0.566
7	12.08 (2.11)	10.74 (1.92)	0.001
8	8.89 (2.2)	9.41 (1.71)	0.194
9	11.43 (2.66)	10.67 (3.05)	0.146
10	10.03 (2.46)	12.09 (1.93)	0.000
11	5.78 (1.34)	6.56 (1.82)	0.007
12	8.14 (2.29)	8.8 (2.54)	0.137
13	5.57 (1.37)	6.27 (1.66)	0.011
14	4.49 (0.87)	5.03 (1.18)	0.003
15	6.27 (2.57)	5.46 (1.5)	0.076
Subscales (COPE)			
I.	55.03 (6.13)	54.9 (5.78)	0.910
II.	41.68 (6.88)	42.79 (5.27)	0.368
III.	43.92 (8.4)	46.13 (7.16)	0.155

Note: *n*—number; *p*—*p*-value based on chi-square test of independence or Student’s *t*-test. 1. Active coping; 2. Planning; 3. Seeking instrumental support; 4. Seeking emotional support; 5. Suppression of competing activities; 6. Turning to religion; 7. Positive reinterpretation and development; 8. Restraint coping; 9. Acceptance; 10. Focus on and venting of emotions; 11. Denial; 12. Mental disengagement; 13. Behavioral disengagement; 14. Substance use; 15. Sense of humor. I. Problem-Focused Coping; II. Emotion-Focused Coping; III. Avoidant Coping. HADS D—Depression categories; HADS A—Anxiety categories; HADS I—Irritability categories.

**Table 2:** Stress coping strategies by parents’ age and gender (*N* = 154).

Strategies (COPE)	Gender		*p*	Age			*p*
	Female (*N* = 132)	Male (*N* = 22)		≤29 (*N* = 51)	30–34 (*N* = 53)	≥35 (*N* = 50)	
1.	11.78 (1.85)	11.82 (2.34)	0.943	11.78 (1.94)	11.51 (2.12)	12.08 (1.66)	0.324
2.	11.65 (2.49)	11.55 (1.84)	0.815	11.14 (2.65)	11.51 (2.45)	12.28 (1.94)	0.050
3	11.79 (2.72)	9.73 (2.66)	0.002	11.12 (3.2)	11.79 (2.48)	11.56 (2.69)	0.463
4	10.78 (3.25)	9.45 (3.17)	0.081	11.14 (3.16)	10.38 (3.18)	10.26 (3.43)	0.340
5	11.26 (2.02)	10.55 (1.92)	0.121	11.04 (2.24)	10.79 (1.85)	11.66 (1.87)	0.080
6	12.03 (3.44)	11.27 (3.17)	0.313	12.25 (3.01)	11.92 (3.55)	11.58 (3.65)	0.611
7	11.12 (2.08)	10.73 (1.8)	0.361	10.43 (2.05)	11.09 (2.12)	11.68 (1.77)	0.008
8	9.35 (1.87)	8.91 (1.66)	0.268	9.35 (1.82)	9.19 (2.03)	9.32 (1.68)	0.892
9	10.91 (2.99)	10.5 (2.87)	0.544	10.47 (3.26)	11.13 (2.93)	10.94 (2.71)	0.511
10	11.67 (2.21)	11.14 (2.42)	0.343	12.27 (1.99)	11.51 (2.31)	10.98 (2.26)	0.013
11	6.45 (1.73)	5.91 (1.8)	0.202	6.53 (1.75)	6.17 (1.58)	6.42 (1.91)	0.560
12	8.69 (2.52)	8.36 (2.36)	0.558	8.47 (2.63)	8.64 (2.52)	8.82 (2.35)	0.783
13	6.15 (1.66)	5.82 (1.33)	0.303	6.16 (1.6)	6.26 (1.56)	5.88 (1.7)	0.468
14	4.83 (1.03)	5.36 (1.59)	0.138	5 (1.08)	4.91 (1.11)	4.8 (1.23)	0.679
15	5.77 (1.91)	5 (1.2)	0.016	5.33 (1.73)	6.06 (2.13)	5.56 (1.54)	0.121
Subscales (COPE)							
I.	55.16 (5.83)	53.55 (5.82)	0.239	53.75 (6.01)	54.09 (6.2)	57.02 (4.71)	0.008
II.	42.79 (5.77)	40.95 (5.01)	0.130	41.96 (5.68)	43.17 (5.98)	42.42 (5.42)	0.552
III.	46.27 (7.43)	41.59 (6.84)	0.006	46.78 (7.31)	45.6 (7.67)	44.38 (7.49)	0.276

Note: *N*—number; *p*—*p*-value based on chi-square test of independence. 1. Active coping; 2. Planning; 3. Seeking instrumental support; 4. Seeking emotional support; 5. Suppression of competing activities; 6. Turning to religion; 7. Positive reinterpretation and development; 8. Restraint coping; 9. Acceptance, 10. Focus on and venting of emotions; 11. Denial; 12. Mental disengagement; 13. Behavioral disengagement; 14. Substance use; 15. Sense of humor; I. Problem-Focused Coping; II. Emotion-Focused Coping; III. Avoidant Coping. Parents’ age was positively correlated with the choice of strategy 7. Positive reinterpretation and development and the score on subscale I. Problem-Focused Coping; however, it was negatively correlated with the choice of strategy 10. Focus on and venting of emotions.

**Table 3:** Spearman’s rank correlations between the choice of stress coping strategies, the level of stress, and parents’ age.

COPE	PSS Score	Age
1	0.109	0.071
2	0.052	0.157
3	−0.036	0.078
4	−0.110	−0.108
5	0.062	0.141
6	−0.020	−0.022
7	−0.199 *	0.253 **
8	−0.065	0.021
9	−0.265 ***	0.006
10	0.362 ***	−0.239 **
11	0.213 **	−0.064
12	0.166 *	0.038
13	0.033	−0.120
14	0.263 ***	−0.122
15	−0.099	0.075
Subscales (COPE)		
I.	−0.056	0.247 **
II.	0.017	−0.025
III.	−0.030	−0.120

Note: *** *p* < 0.001, ** *p* < 0.01, * *p* < 0.05. 1. Active coping; 2. Planning; 3. Seeking instrumental support; 4. Seeking emotional support; 5. Suppression of competing activities; 6. Turning to religion; 7. Positive reinterpretation and development; 8. Restraint coping; 9. Acceptance; 10. Focus on and venting of emotions; 11. Denial; 12. Mental disengagement; 13. Behavioral disengagement; 14. Substance use; 15. Sense of humor. I. Problem-Focused Coping; II. Emotion-Focused Coping; III. Avoidant Coping.

**Table 4:** Logistic regression analysis—association between high level of PSS and high level of COPE strategies (above second tertile of distribution of individual COPE strategies score).

COPE	Model 1	Model 2	Model 3
1	0.82 (0.38; 1.79)	0.84 (0.39; 1.85)	0.92 (0.41; 2.11)
2	3.16 (1.35; 8.13) *	3.17 (1.34; 8.25) *	3.45 (1.43; 9.16) **
3	1.04 (0.48; 2.32)	1.00 (0.44; 2.31)	1.00 (0.44; 2.33)
4	0.73 (0.34; 1.55)	0.66 (0.30; 1.42)	0.67 (0.30; 1.45)
5	1.03 (0.49; 2.21)	0.99 (0.45; 2.16)	1.01 (0.46; 2.23)
6	1.50 (0.69; 3.41)	1.49 (0.67; 3.43)	1.45 (0.64; 3.35)
7	0.20 (0.08; 0.45) ***	0.18 (0.07; 0.44) ***	0.19 (0.07; 0.46) ***
8	1.46 (0.69; 3.21)	1.43 (0.66; 3.19)	1.37 (0.62; 3.10)
9	0.93 (0.44; 2.00)	0.89 (0.41; 1.94)	0.94 (0.43; 2.08)
10	3.95 (1.59; 11.40) **	3.72 (1.48; 10.85) **	4.17 (1.61; 12.49) **
11	2.56 (1.14; 6.13) *	2.49 (1.10; 6.03) *	2.53 (1.11; 6.17) *
12	1.61 (0.73; 3.72)	1.62 (0.73; 3.77)	1.59 (0.70; 3.74)
13	2.77 (1.20; 7.06) *	2.74 (1.17; 7.06) *	2.72 (1.15; 7.09) *
14	2.96 (1.35; 6.83) **	2.93 (1.31; 6.86) *	3.36 (1.46; 8.18) **
15	0.89 (0.42; 1.92)	0.95 (0.43; 2.10)	1.00 (0.45; 2.27)
Subscales (COPE)			
I.	0.52 (0.24; 1.12)	0.48 (0.21; 1.08)	0.47 (0.20; 1.07)
II.	1.41 (0.64; 3.31)	1.39 (0.61; 3.31)	1.39 (0.61; 3.33)
III.	1.17 (0.54; 2.61)	1.04 (0.46; 2.38)	1.04 (0.46; 2.39)

Note: Results are presented via OR (95% CI) of high-level score in COPE strategies (T3 vs. T2 + T1) for high-level PSS compared with low/average level; *** *p* < 0.001, ** *p* < 0.01, * *p* < 0.05. Model 1 includes the following: PSS level (low/average, high), place of residence (village, city), and education (vocational/secondary, high). Model 2 includes the following: PSS level (low/average, high), place of residence (village, city), education (vocational/secondary, high), age categories (29−, 30–34, 35+), and sex (female, male). Model 3 includes the following: PSS level (low/average, high), place of residence (village, city), education (vocational/secondary, high), age categories (29−, 30–34, 35+), sex (female, male), correction (complete, incomplete, none), and period when defect was detected (in prenatal phase or after giving birth). Note: 1. Active coping; 2. Planning; 3. Seeking instrumental support; 4. Seeking emotional support; 5. Suppression of competing activities; 6. Turning to religion; 7. Positive reinterpretation and development; 8. Restraint coping; 9. Acceptance; 10. Focus on and venting of emotions; 11. Denial; 12. Mental disengagement; 13. Behavioral disengagement; 14. Substance use; 15. Sense of humor. I. Problem-Focused Coping; II. Emotion-Focused Coping; III. Avoidant Coping.

## Data Availability

The data presented in this study are available on request from the corresponding author. The data are not publicly available due to specific ethical and privacy considerations.
